# Experimental Study on Panic during Simulated Fire Evacuation Using Psycho- and Physiological Metrics

**DOI:** 10.3390/ijerph19116905

**Published:** 2022-06-05

**Authors:** Kaifeng Deng, Meng Li, Guanning Wang, Xiangmin Hu, Yan Zhang, Huijie Zheng, Koukou Tian, Tao Chen

**Affiliations:** 1 Department of Engineering Physics, Institute of Public Safety Research, Tsinghua University, Beijing 100084, China; meng-li18@mails.tsinghua.edu.cn (M.L.); wgn19@mails.tsinghua.edu.cn (G.W.); hxm20@mails.tsinghua.edu.cn (X.H.); zhang-y21@mails.tsinghua.edu.cn (Y.Z.); zhenghj19@mails.tsinghua.edu.cn (H.Z.); tiankk20@mails.tsinghua.edu.cn (K.T.); 2 Anhui Province Key Laboratory of Human Safety, Hefei 230601, China; 3 Beijing Key Laboratory of Comprehensive Emergency Response Science, Beijing 100084, China

**Keywords:** public safety, fire evacuation, panic, eye movement, haemodynamics, statistical analysis

## Abstract

Under circumstances of fire, panic usually brings uncertainty and unpredictability to evacuation. Therefore, a deep understanding of panic is desired. This study aims to dig into the underlying mechanism of fire evacuation panic by measuring and analysing psycho- and physiological indicators. In the experiment, participants watched a simulated train station within which three sets of stimuli were triggered separately. Eye movement and brain haemodynamic responses were collected during the watch, while questionnaires and interviews of emotions were conducted after. The analysed physiological indicators include the amplitude of pupil dilation, the time ratios of fixation and saccade, the binned entropy of gaze location, and the brain activation coefficients. The results of this research indicate that fire evacuation panic can be broken down into two elements. (1) Unawareness of situation: less knowledge of the situation leads to a higher level of panic; (2) Intensity of visual stimulation: the panic level is escalated with increased severity of fire that is perceived.

## 1. Introduction

Evacuation is a major tool in the handling of emergency events. When placed into the wider background of risk management, evacuation plays a critical role in risk reduction. Risk management itself concerns numerous disciplines, among which are mining [1,2,3], earthquakes [4], marine disasters [5], fires [6], etc. Russo and Vitetta presented the formulation of risk over an intensity range of emergency event, an area, and a time period, as in Equation (1) [7], where *R* represents the overall risk, *P* the probability of an emergency event, *V* the vulnerability, and *N* the exposure. *P*, *V*, and *N* can either be constants, or be non-constants to be integrated over the intensity range, the area and the time period. Evacuation, in this sense, is a possible intervention to reduce the exposure component *N*, hence reducing the risk *R* [7,8].

(1) R=PVN

The evacuation process is influenced by a very wide range of components. It is apparent that the environment where the evacuation takes place exerts significant influence on the process. Related to this concept are various components that present the properties of the environment, including the building scale [9], the lighting [10], the emergency signs [11], the rescuer allocation [12], the bottlenecks [13], the surrounding crowd [14], the modal transitions [15], etc. Another group of components concerns the properties of the evacuees. Mental states certainly do influence the evacuation, including emotions [16], preparedness [17], the socio-cultural backgrounds [18], etc., not to mention the obvious physical characteristics, including age, fitness, gender, etc.

Indoor fire (henceforth referred to as fire) is one of the major causes for building evacuations and often brings casualties. On 8 December 1994, a fire took 325 lives in a theatre in Karamay, Xinjiang [19], which remains to this day one of the most notorious disasters in China. On 21 December 2017, a sports centre fire in Korea that killed 29 and injured 29 caught the world’s attention by causing disturbance to the torch relay of Pyeongchang Winter Olympics [20]. Statistics show that through 2015 to 2019 in China, there were around 300,000 indoor fires annually, and the death toll averaged 1523 per year [20].

The confrontation with fire usually induces complicated psychological responses [21]. For convenience of expression in this paper, the term ‘panic’, as in a general sense, is hereby and thereafter adopted to describe these responses, although the precise definition of ‘panic’ may be ambiguous [22]. The complex nature of human psychology brings uncertainty and unpredictability to fire evacuations. Many researches have so far been dedicated to this issue. Multiple studies have integrated the impact of panic into pedestrian simulation models with panic parameters [23,24,25,26]. However, a deeper understanding of the mechanism of panic is in many cases lacking, due to insufficiency with empirical or experimental data concerning panic during evacuation. Psychological surveys were conducted in some studies as a method to understand panic [27,28,29], but not many of them were further validated by metrics of more objectivity and quantitativeness. Physiology could function as such metrics, as psychological responses are often manifested in the form of physiological signals [30,31]. Hence, it is of particular interest to find out the way the physiological signals evolve under various circumstances of fire. For reasons of safety and convenience, a simulated fire could be utilised instead of a real one. The validity of virtual simulations has been scrutinised by numerous researchers, and many conclude that the experiences in virtual and real environments are comparable [32,33,34]. This study aims to dig into the mechanism of evacuation panic by setting up an experiment that simulates fire, conducting psychological questionnaires and interviews, and further quantitatively validating them with physiological measurements.

**Figure 1:**
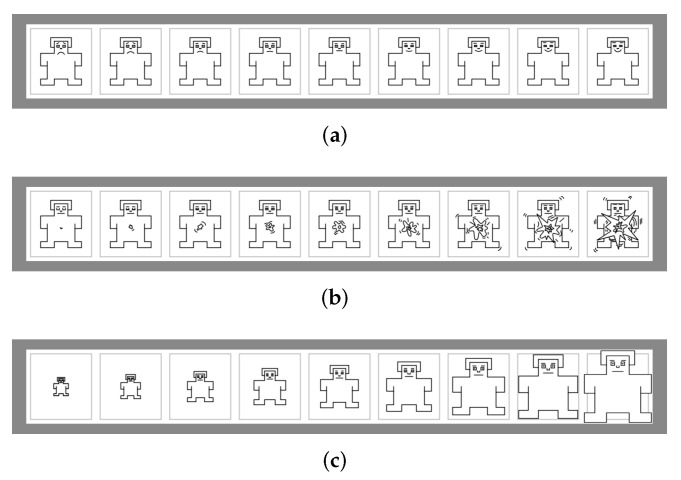
Self-Assessment Manikin (SAM). (**a**) Valence, (**b**) Arousal, (**c**) Dominance.

The psychological questionnaires are in this study composed of two sets of emotion scales. The basic emotions (sadness, happiness, fear, anger, surprise, disgust) are considered psychologically distinct and universally recognised [35,36]. The Self-Assessment Manikin (SAM) [37] is a pictographic scale to assess emotion in three independent dimensions which are valence (positive or negative feeling), arousal (excitation or boredom), and dominance (how much a person feels in control of a situation). The human-like pictorial representation is instinctive and may direct to further reliable decision on perceived emotion [38]. Figure 1 shows the three dimensions of SAM [39,40], where in each of the rows, the 9 pictures correspond to the scores 1–9 from left to right. Both the basic emotions and SAM are designed as 9-point scales in this study.

A wide range of physiological signals have been used in various studies for multiple purposes. Lin et al. measured the heart rate and blood pressure of metro passengers who were confronted with a virtual fire, but evacuation was not involved [41]. Martens et al. simulated an elevator and used the height as the stressor, collected multiple physiological signals (salivary cortisol and alpha-amylase, blood pressure, pulse, skin conductance) of participants, and explored the impact on working memory [42]. Shi et al. created a virtual environment with an industrial maintenance task, studied the evolution of eye movement and haemodynamic responses with and without smoke present, and built their links to the accuracy of the task [43]. In this research, we select the haemodynamic responses and eye movement as the physiological metrics to evaluate and reveal the underlying mechanism of fire evacuation panic. Functional Near-Infrared Spectroscopy (fNIRS) measures the intensity of light between every pair of optical emitter and detector (or channel) [44], which is then converted by the modified Beer–Lambert Law [45] into the concentration changes of brain haemoglobin. Two groups of light with different wavelengths (845/757 nm) are emitted and detected at every channel, and they lead to the concentrations of both oxygenated and deoxygenated haemoglobin. An eye tracker examines the position and size of pupils throughout the entire experiment, from which more sophisticated metrics are then obtained and output, including but not limited to fixation, saccade, and gaze coordinate [46].

## 2. Materials and Methods

### 2.1. Setup of Experiment

**Figure 2:**
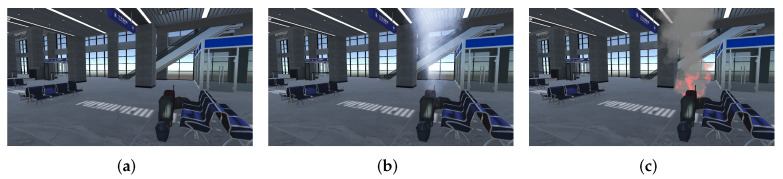
Stimuli. (**a**) Alarm, (**b**) Smoke, (**c**) Flame.

The experiment consist of 3 sets of stimuli, each followed by a simulated evacuation process, in a virtual train station. The stimuli are specified as follows and depicted in Figure 2.

1. Alarm only (henceforth referred to as ‘Alarm’);
2. Smoke with alarm (henceforth referred to as ‘Smoke’);
3. Flame with smoke and alarm (henceforth referred to as ‘Flame’).

**Figure 3:**
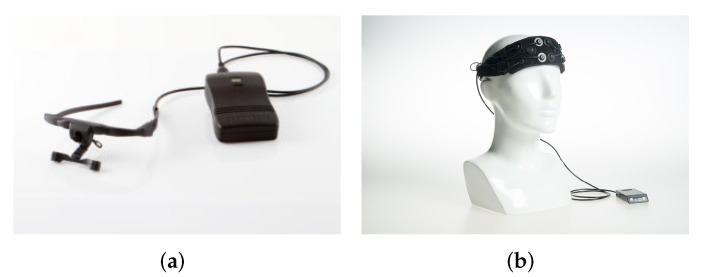
Devices. (**a**) Dikablis Glasses 3, (**b**) Artinis OctaMon.

**Figure 4:**
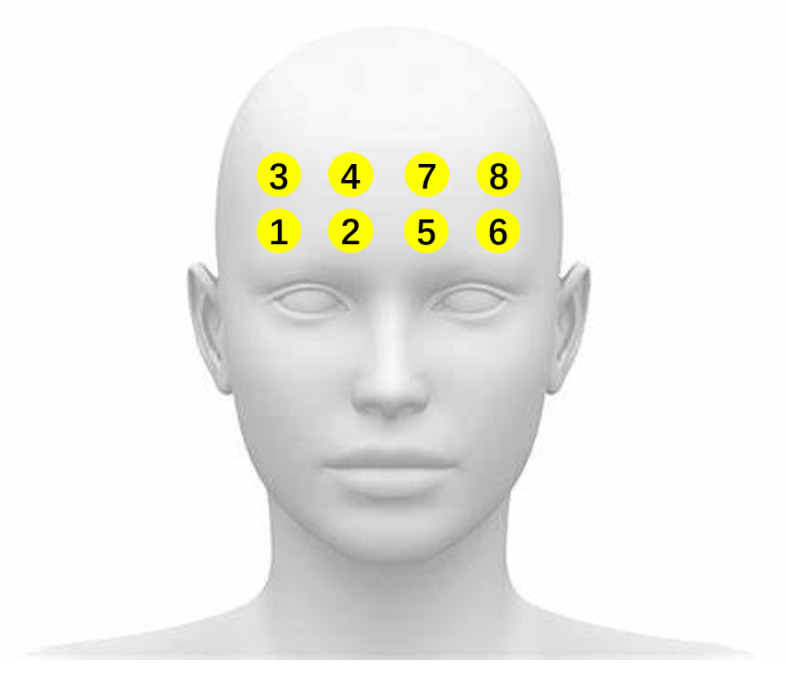
Channel order of fNIRS.

A set of wearable eye tracker *Dikablis Glasses 3* [47] was used to measure the behaviour of the eyes at 60 Hz, and an 8-channel *Artinis OctaMon* fNIRS device [48] was used to measure the concentration of oxy- and deoxy-haemoglobin, sampling at 10 Hz, at 8 distinct positions of the forehead. Figure 3 shows the respective devices. The indexing order of fNIRS channels is demonstrated as in Figure 4.

**Figure 5:**
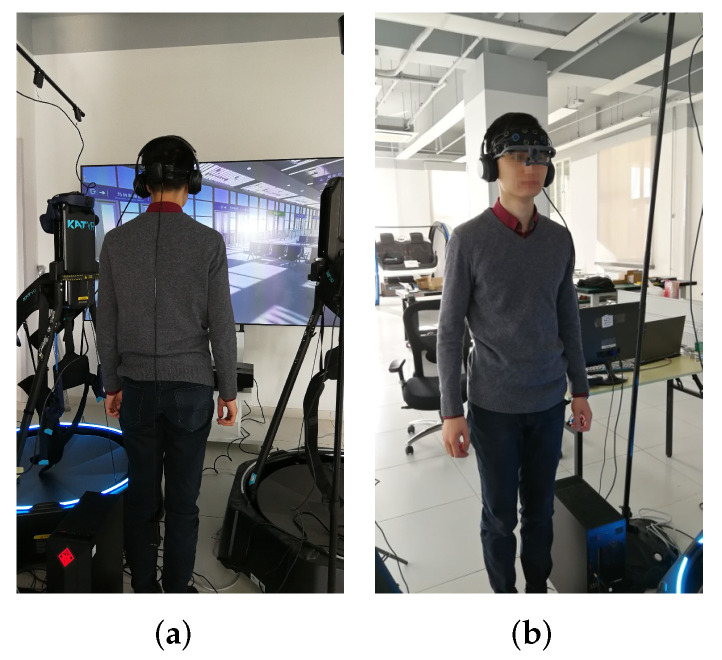
Setup example. (**a**) Back, (**b**) Front.

A total number of 56 healthy adults (gender: male = 34, female = 22; age: μ=24.16, σ=4.44, max=49, min=19) consensually participated in this experiment. Among them, 15 were undergraduate students from Tsinghua University, 37 were graduate students, and 4 were faculty. None of them reported any experience of real-world fire events. The participants were unaware of the stimuli beforehand for the sake of authenticity. They were required to experience the stimuli and evacuation processes standing 1.8 m in front of an 88-inch screen with a set of earphones, while wearing physiological devices. An example of the setup can be found in Figure 5. After all 3 stimulus-evacuation rounds, the participants were asked to recall their emotions throughout the experience and fill out the basic emotion and SAM scales. Both were designed as 9-point scales.

### 2.2. Procedure of Experiment

For each of the participants, the entire experiment was conducted in 3 phases.

#### 2.2.1. Preparation

The participants were required to fill out basic information and put on wearable devices. Calibration of eye tracker was also completed during this phase.

#### 2.2.2. Simulation

**Figure 6:**
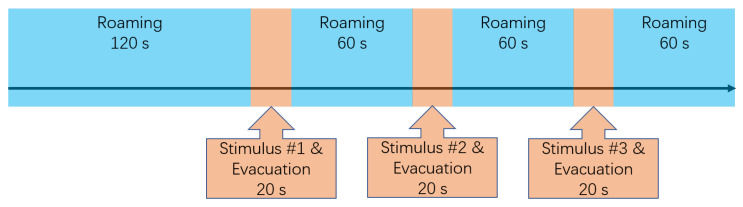
Procedure of simulation phase.

The participants were told to immerse themselves into the virtual context, and not to deliberately touch the idea that this was a virtual simulation. Before any stimuli were applied, the participants roamed the virtual train station for 2 min (henceforth referred to as ‘Roaming’). The purpose of the roaming period is to let the participants familiarise themselves with the environment, and to provide a baseline for the stimuli later on. The roaming period was subsequently followed by 3 stimulus-evacuation rounds. During each round, the participants experienced one specific stimulus and evacuated to the exit, which took approximately 20 s. They roamed the environment for 1 min after each stimulus-evacuation round, which served as a buffer. Figure 6 describes this procedure.

To eliminate the impact of the order of stimuli, they were organised in 3 different sequences.

1. Alarm-Smoke-Flame (26 participants);
2. Smoke-Flame-Alarm (15 participants);
3. Flame-Alarm-Smoke (15 participants).

#### 2.2.3. Questionnaires and Verbal Description

During this phase, the participants recollected their emotions throughout the simulation phase and completed the basic emotion and SAM scales. The meanings of the scales were explained to the participants in detail to ensure the validity of the emotion scores that were collected. They were required to recall their emotions in correspondence to ‘Roaming’, ‘Alarm’, ‘Smoke’, and ‘Flame’, respectively, for every question in the scales. The participants were also invited to add comments if they had any, usually on which stimulus they considered the strongest and why they felt that way.

## 3. Results

### 3.1. Subjective Account

Of the 56 participants, 46 considered ‘Alarm’ as the strongest stimulus, 9 chose ‘Flame’, while the remaining 1 participant chose ‘Smoke’. Thirty-three participants specifically mentioned that, to various degrees, their unawareness of the situation led to the greatest panic with regard to the ‘Alarm’ scenario, i.e., they did not know what the alarm was for, whilst the situations were easily understood in the other two scenarios. Fifty-one participants described the visual effect of ‘Flame’ as more stimulating than that of ‘Smoke’, whereas none mentioned any difference between these two scenarios regarding unawareness of situation. This indicates that the responses of participants in this study can be attributed to two elements, listed as below:

1. Unawareness of situation, which may cause the responses to ‘Alarm’ to be greater than those to ‘Smoke’ and ‘Flame’;
2. Intensity of visual stimulation, which may cause a stronger response to ‘Flame’ than to ‘Smoke’, however may have no impact on ‘Alarm’.

It is also worth mentioning that only 2 of the 56 participants stated a clear sense of falsehood regarding the virtual simulation, which ensures the validity of the experiment.

### 3.2. Emotion Scales

For every term *x* in the scales (namely *x* represents sadness, happiness, fear, anger, surprise or disgust for the basic emotion scale, and valence, arousal or dominance for the SAM scale), the participants were required to evaluate $x_{r}$ for ‘Roaming’, $x_{a}$ for ‘Alarm’, $x_{s}$ for ‘Smoke’, and $x_{f}$ for ‘Flame’, respectively. Setting $x_{r}$ as baseline, the shift of *x* is used as an indicator for the impact of the stimuli on emotion. Namely:

(2) $$\left\{\begin{array}{cc} \; & \Delta x_{a}=x_{a}-x_{r}\\ \; & \Delta x_{s}=x_{s}-x_{r}\\ \; & \Delta x_{f}=x_{f}-x_{r} \end{array}\right.$$

The *t*-test [49] is subsequently conducted on Δx between every two of the three scenarios to determine the significance in the difference of mean values.

**Figure 7:**
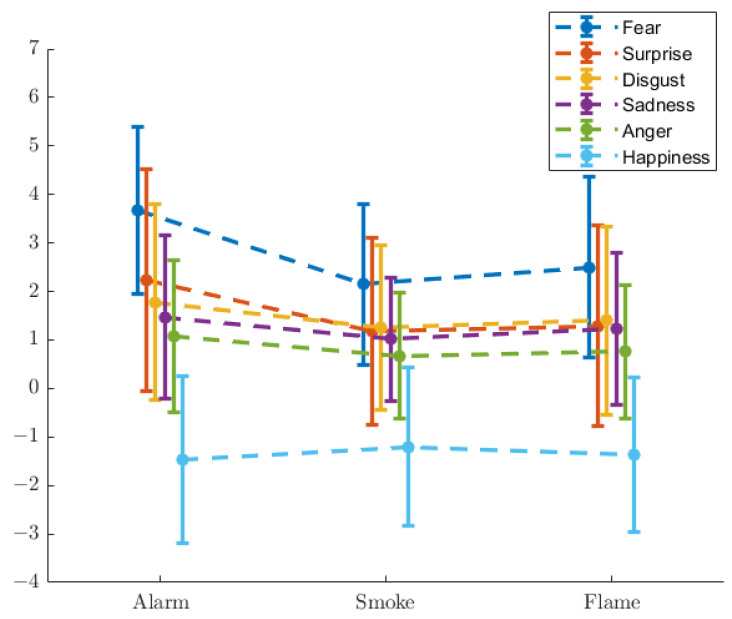
Shifts in basic emotion scores.

Figure 7 demonstrates the shifts of basic emotion scores (Note that the dotted lines in Figure 7 do not represent any functions. They are merely intended for a clearer illustration. This applies to similarly styled figures throughout this paper.), as defined in Equation (2). Table 1 and Table 2 exhibit the mean values and standard deviations, respectively, of basic emotion shifts. Table 3 shows the *p*-values from the *t*-test, where the numbers are bolded for p<0.05.

The values of basic emotion shifts indicate that fear and surprise are the most relevant two emotions with regard to panic. In both cases, ‘Alarm’ induces the largest shifts, in accordance with the participants’ narration. The difference between ‘Smoke’ and ‘Flame’, with respect to surprise, does not reach the threshold of significance. It is then instinctive that surprise corresponds to unawareness of situation, as introduced in Section 3.1.

**Figure 8:**
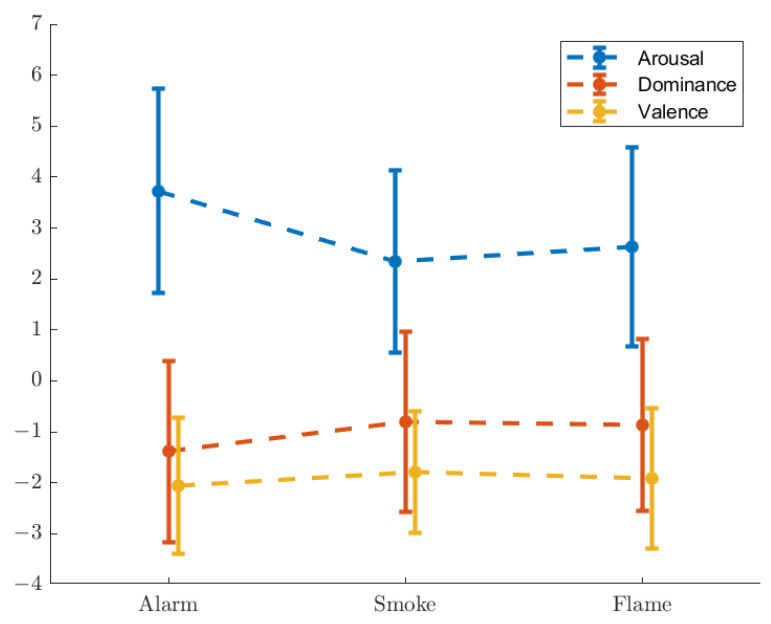
Shifts in SAM scores.

The shifts of SAM scores are as Figure 8. Table 4, Table 5 and Table 6 exhibit the mean values, standard deviations, and *p*-values of SAM shifts.

It is seen that arousal and dominance are more relevant in this study than valence. Similar to basic emotions, ‘Alarm’ causes the greatest shifts. No significant difference is detected between ‘Smoke’ and ‘Flame’ with respect to dominance, indicating that dominance reflects unawareness of situation mentioned in Section 3.1.

### 3.3. Eye Tracking

#### 3.3.1. Pupil Area

**Figure 9:**
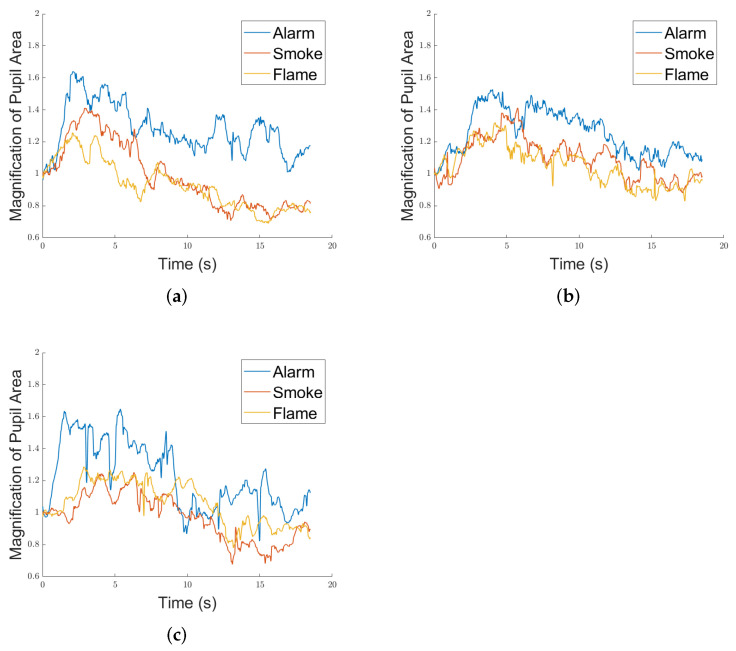
Three typical cases of pupil dilation. (**a**) Case 1, (**b**) Case 2, (**c**) Case 3.

Typically, the pupils dilate with panic [50]. For every time point, the average area (denoted as $A_{t}$, at the *t*-th time point) of the left and right eyes is taken as the indicator for this part of study. The starting 10 time points are set as the baseline for magnification (denoted as $M_{t}$, at the *t*-th time point), i.e., all pupil area values of the 20-s period are divided by the mean value of the first 10 time points. Equation (3) offers a formulaic definition of this metric. There are short periods of time when pupils are not detected due to blinks or other disturbances. The pupil area of these periods is linear-interpolated since they are typically very short. Figure 9 shows 3 typical cases of pupil dilation, each from a separate participant, when they went through the 3 types of stimuli and the following evacuation processes. The vertical axes represent $M_{t}$.

(3) $$M_{t}=\frac{A_{t}}{\frac{1}{10}\sum_{i=1}^{10}A_{i}}$$

It is observed that in a general sense, the area of pupil increases shortly after the stimuli are applied, and later decreases towards the end of evacuation. Hence, pupil dilation could serve as a representation for the participants’ psychology. The amplitudes of pupil dilation, i.e., the maximal magnification of pupil area throughout the stimulus–evacuation rounds, are therefore recorded and *t*-tested between every two of the three scenarios.

**Figure 10:**
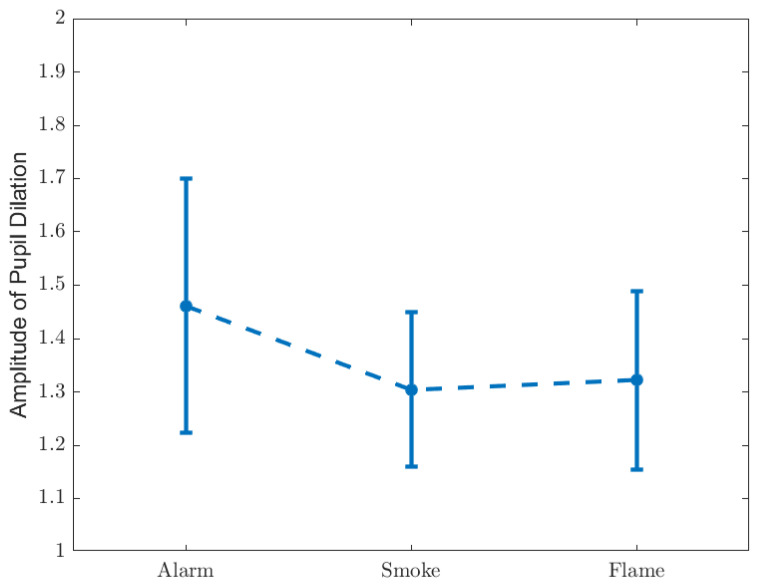
Amplitude of pupil dilation.

The amplitude of pupil dilation, as defined above, is shown in Figure 10, and listed in Table 7 and Table 8.

It is observed that ‘Alarm’ causes the greatest pupil dilation, whilst no significant gap is found between ‘Smoke’ and ‘Flame’, suggesting that pupil dilation is related only to unawareness of situation.

#### 3.3.2. Fixation and Saccade

Fixation and saccade are a widely used pair of indicators related to attention [51,52]. The eye tracker outputs for each eye at every time point two boolean variables that represent fixation and saccade separately. These two boolean variables are opposite to each other, except for the cases of blinks and other possible disturbances. The time ratios of fixation and saccade, hereby represented respectively by $r_{\mathrm{fixation}}$ and $r_{\mathrm{saccade}}$, are calculated by Equations (4) and (5), where $F_{t}$ and $S_{t}$ are the boolean variables for fixation and saccade at the *t*-th time point, and *N* denotes the total number of time points. Equation (6) explains the $\left[\cdot \right]$ function. For every stimulus-evacuation round and ‘Roaming’ period, the time ratios of fixation and saccade are calculated by taking the average of both eyes, and *t*-tested.

(4) $$\begin{array}{c} r_{\mathrm{fixation}}=\frac{\sum_{t}\left[F_{t}\right]}{N} \end{array}$$

(5) $$\begin{array}{c} r_{\mathrm{saccade}}=\frac{\sum_{t}\left[S_{t}\right]}{N} \end{array}$$

(6) $$\left[x\right]=\left\{\begin{array}{c} 1,\mathrm{for}\;x\;\mathrm{is}\;\mathrm{true}\\ 0,\mathrm{for}\;x\;\mathrm{is}\;\mathrm{false} \end{array}\right.$$

**Figure 11:**
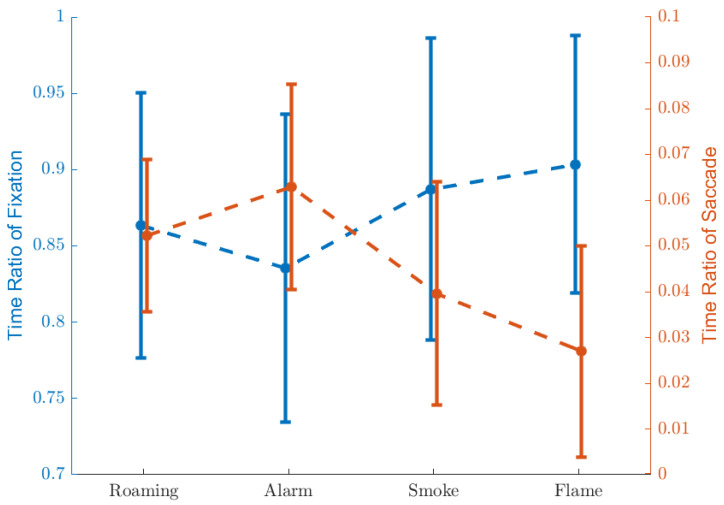
Time ratios of fixation and saccade.

Figure 11 illustrates the time ratios of fixation and saccade, defined in Equations (4) and (5). The numerics are listed in Table 9, Table 10 and Table 11.

Compared to ‘Roaming’, ‘Alarm’ causes less fixation and more saccade, indicating that participants were spending longer searching around for clues. ‘Smoke’ and ‘Flame’, in the contrary, cause more fixation and less saccade, suggesting more attention being paid to the visual effects. Specially, the saccade ratio is even lower in the case of ‘Flame’ than ‘Smoke’, which could relate to the intensity of visual stimulation. No significant difference was found between the fixation ratios of ‘Flame’ and ‘Smoke’, however the *p*-value in this case is just on the edge of the threshold. Given that the boolean variables of fixation and saccade are in most cases opposite (or equivalently, a near-perfect negative correlation), we believe that the near-significant insignificance (p=0.056) could be due to random fluctuation, and hence that the time ratio of fixation actually has a relation with the intensity of visual stimulation.

#### 3.3.3. Gaze

Entropy describes randomness or uncertainty, defined as Equation (7) [53]:

(7) $$S=-\int_{x}p\left(x\right)\log p\left(x\right)dx$$

In this case, the view plane is divided into 10×10-pixel grids, or bins, on which the binned entropy is calculated to measure the randomness or uncertainty of a temporal series of gaze positions [54]. Let *i* denote the index of bins, *N* denote the total number of time points, and $n_{i}$ denote the number of time points where the gaze position falls in the *i*-th bin. The binned entropy thus follows:

(8) $$S_{\mathrm{binned}}=-\sum_{i}\frac{n_{i}}{N}\log\frac{n_{i}}{N}=\log N-\frac{1}{N}\sum_{i}n_{i}\log n_{i}$$

The binned entropy is henceforth calculated for every stimulus-evacuation round as well as ‘Roaming’ period, and *t*-tested.

**Figure 12:**
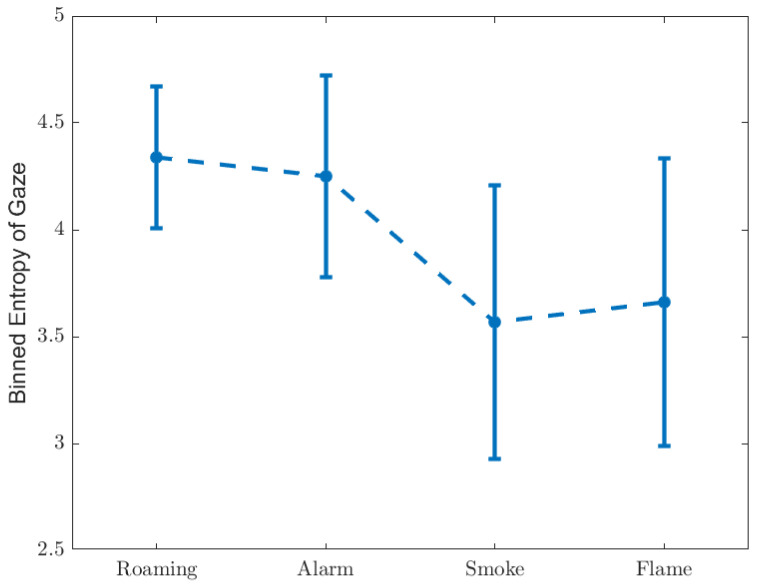
Binned entropy of gaze.

The binned entropy of gaze location, defined as Equation (8), is shown in Figure 12. Table 12 and Table 13 list the statistical results.

Although the time ratio of fixation increases in the case of ‘Alarm’ as shown in Figure 11, the binned entropy of gaze coordinate stays level in comparison with ‘Roaming’, indicating that the spatial randomness does not go up despite more saccade. The binned entropy decreases in the cases of ‘Smoke’ and ‘Flame’, however without significant gap between these two scenarios. This suggests that awareness of situation co-occurs with a less random gaze pattern, and that the intensity of visual effect has very little impact on the spatial randomness of gaze.

### 3.4. fNIRS

**Figure 13:**
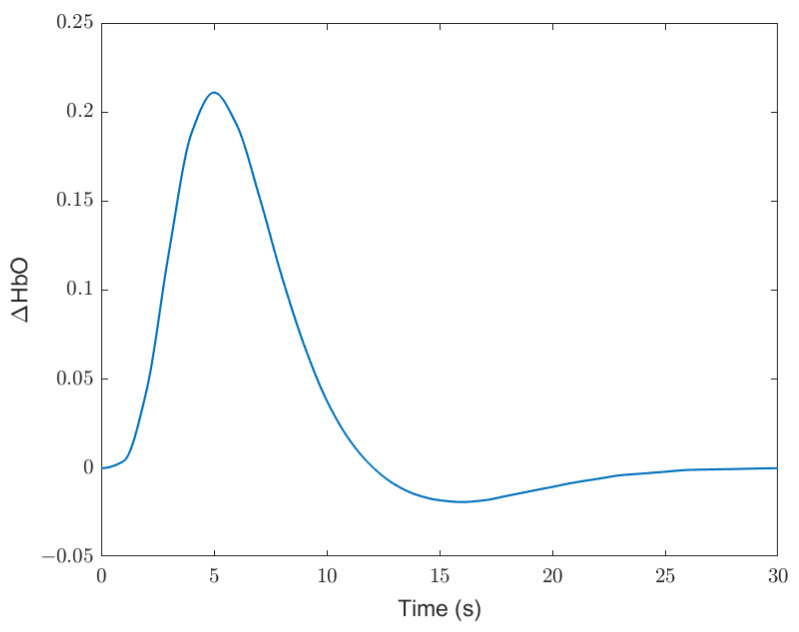
Canonical haemodynamic response function (HRF).

The change of concentration of oxyhaemoglobin (ΔHbO) is selected in this study as the indicator of brain activation. The ΔHbO typically spikes approximately 5 s after a stimulus, drops to a minima at round 16 s, and then returns to homeostatic level at 30 s. The canonical haemodynamic response function (HRF) depicts a standard case of this process, shown in Figure 13 [55].

Since the stimuli in this study were applied to the participants nearly instantaneously, it is therefore reasonable to assume the temporal distribution of a stimulus as a Kronecker δ-function $\delta_{t,t_{i}}$, where $t_{i}$ denotes the timestamp of the *i*-th stimulus. By convolving it with the HRF, one can obtain the theoretical response, which in this case is $\delta_{t,t_{i}}⊗\mathrm{HRF}$. The resulting ΔHbO signal is a linear combination of the responses induced by all three stimuli as Equation (10), where $\beta_{i}$ denotes the activation coefficient with regard to the *i*-th stimulus.

(9) $$f_{i}\left(t\right)=\delta_{t,t_{i}}⊗\mathrm{HRF}$$

(10) $$\Delta \mathrm{HbO}=\sum_{i=1}^{3}\beta_{i}f_{i}\left(t\right)=\sum_{i=1}^{3}\beta_{i}\delta_{t,t_{i}}⊗\mathrm{HRF}$$

**Figure 14:**
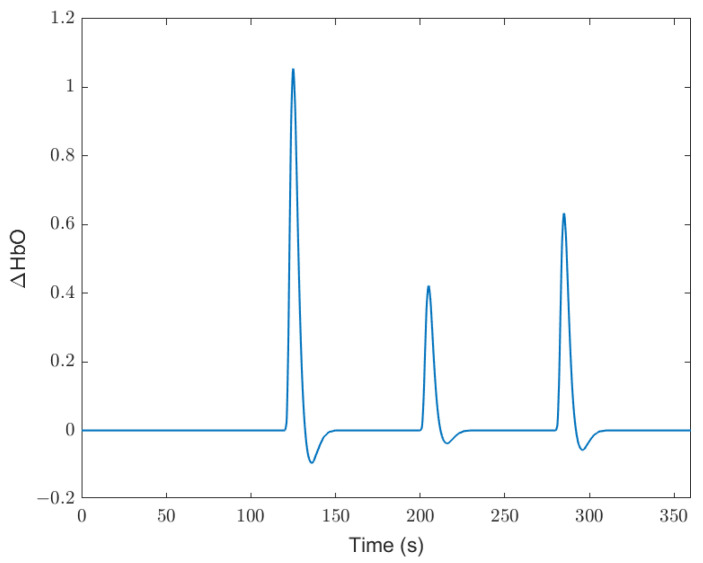
Ideal response signal.

Figure 14 shows an ideal response signal.

**Figure 15:**
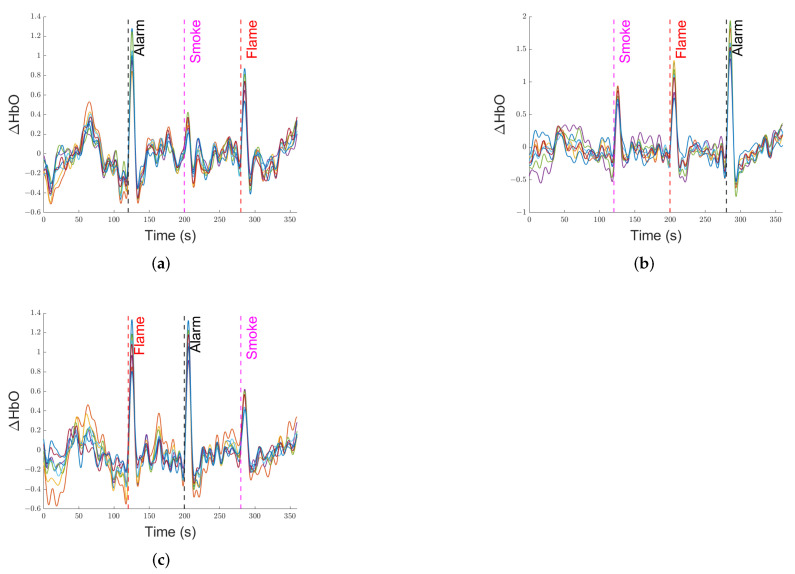
Three cases of filtered ΔHbO signals. (**a**) Case 1, (**b**) Case 2, (**c**) Case 3.

The signal, before further analysis, is fed through a 0.01–0.1 Hz bandpass filter to remove most physiological noise and slow-wave drift [56]. Figure 15 demonstrates three cases of filtered ΔHbO signals, where each of the curves corresponds to a separate channel.

The goal here is to solve the activation coefficient β, defined in Equation (10). It is clarified in Figure 6 that every adjacent two stimuli are set 80 s apart, whereas the HRF lasts 30 s. Therefore, every stimulus can be dealt with separately. Let $\vec{y_{j}}$ denote an N×1 column vector that represents the ΔHbO signal of the *j*-th channel, where *N* denotes the number of time points. $\vec{f_{i}}$ is exactly the same as $f_{i}\left(t\right)$ in Equation (9), written as an N×1 column vector. $\beta_{ij}$ represents the activation coefficient with respect to the *i*-th stimulus and the *j*-th channel. Equation (10) can hence be rewritten as the following equation, where ε denotes noise.

(11) $$\vec{y_{j}}=\beta_{ij}\vec{f_{i}}+\varepsilon$$

The least squares estimation of $\beta_{ij}$ is thus obtained through Equation (12) [57] ($\hat{\beta_{ij}}$ stands for an estimation for $\beta_{ij}$, due to the elimination of the noise term):

(12) $$\hat{\beta_{ij}}=\frac{{\vec{f_{i}}}^{T}\vec{y_{j}}}{{\vec{f_{i}}}^{T}\vec{f_{i}}}$$

For every channel of fNIRS, the least squares estimation of $\beta_{ij}$ is computed following Equation (12) and *t*-tested.

**Figure 16:**
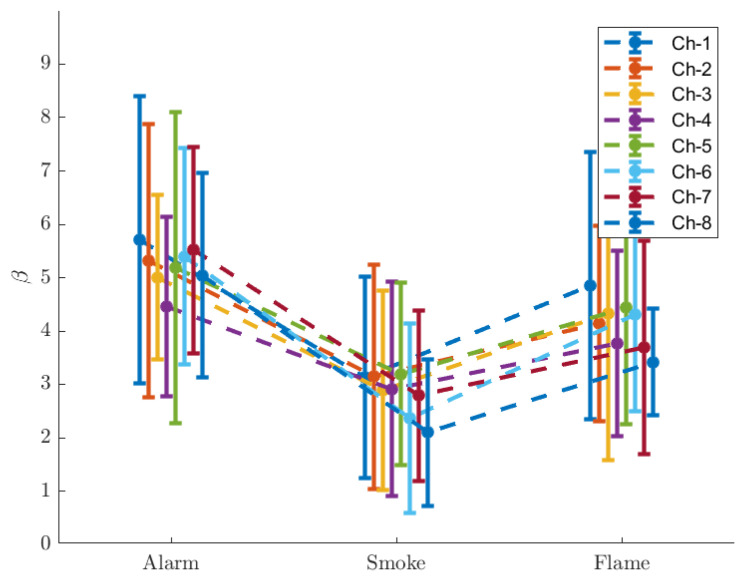
Activation coefficient.

The brain activation coefficients ($\hat{\beta }$ as defined in Equation (12)) for every channel and every stimulus are demonstrated in Figure 16, and exhibited in Table 14, Table 15 and Table 16.

The brain is for every channel most activated with ‘Alarm’, and least activated with ‘Smoke’. The results suggest that brain activation is related both to the unawareness of situation (a greater escalation with ‘Alarm’), and to the intensity of visual stimulation (a greater escalation with ‘Flame’). The differences are determined as insignificant for channels 1, 3 and 5 between ‘Alarm’ and ‘Flame’. In these cases, the unawareness of situation and a more intense visual simulation might induce coincidentally similar increases with regard to brain activation.

## 4. Discussion

This study explores the mechanism of panic during evacuation through an experiment that simulated three different stimuli (namely ‘Alarm’, ‘Smoke’, and ‘Flame’). The participants wore the fNIRS device and eye tracker when they experienced the simulated processes of stimuli and evacuations, for the purpose of measuring the change of haemoglobin concentration and eye data. After the simulation, participants evaluated their emotion changes throughout their experience, and filled out the basic emotion and SAM scales. They were also encouraged to provide any subjective description regarding their feelings on the stimulations.

The panic that the participants experienced is, according to them, attributed to two factors. The first is unawareness of situation with respect to ‘Alarm’ when they could not make sense of what was happening, presumably causing a higher level of panic with ‘Alarm’ than with ‘Smoke’ and ‘Flame’. The other is the intensity of visual stimulation, or in other words the severity that was perceived, presumably causing a higher level of panic with ‘Flame’ than with ‘Smoke’. This is confirmed by the emotion scales, where the scores of fear and arousal represent the overall panic (‘Alarm’ > ‘Flame’ > ‘Smoke’), and the scores of surprise and dominance represent unawareness of situation (‘Alarm’ > ‘Flame’ ≈ ‘Smoke’).

This is also supported by physiological data from this study. Regarding eye tracking data, we analyse the amplitude of pupil dilation, the time ratios of fixation and saccade, as well as the binned entropy of gaze location. It turns out that unawareness/awareness of situation is particularly manifested by the amplitude of pupil dilation and the binned entropy of gaze. Unawareness is related to greater pupil dilation, although does not increase the binned entropy of gaze. Awareness, however, does decease the binned entropy of gaze, as well as being linked to less pupil dilation. The time ratios of fixation and saccade are related to both factors in a way that unawareness increases saccade time and awareness does the opposite, whilst a more intense visual stimulation relates to less saccade and more fixation. The brain activation coefficient ($\hat{\beta }$), calculated from the oxyhaemoglobin data, is chosen for the analysis of haemodynamics. Consistent trends are found for all 8 channels: that unawareness and more intense visual stimulations are both linked with higher $\hat{\beta }$’s, and that unawareness causes even greater increases in $\hat{\beta }$ for 5 out of the 8 channels.

All the findings of this study being listed, there are still issues that need to be addressed by future researches. Since the age and background distributions of participants of this study are biased (mostly 20- to 28-year-old students), future experiments need to be conducted on a wider variety of age groups and backgrounds. There are also possibilities of experimenting with more complicated and refined environment settings as well as stimulations. Specially, this study utilises only visual and auditory information. It would be greatly helpful with regard to the sense of immersion if there is a proper way to apply other sensory stimulations, e.g., smell and heat. This could extend the second sub-element to further stimulations other than visual ones. There are also further variables that may influence the psycho- and physiological evolution during an evacuation (e.g., the capacity of the building, the presence of bottlenecks and queues) that need to be experimented with in the future. It is also noted that this study focuses on the psycho- and physiological dynamics of single persons, hence future researches could be dedicated to the impact of multi-person interactions on panic.

## 5. Conclusions

It is found in this study that panic during fire evacuation is ascribed to unawareness of situation and the intensity of visual stimulation. This is consistent with multiple psycho- and physiological indicators that are measured during the experiment. These indicators can be categorised into two groups: one that is linked solely with unawareness of situation, and the other that is linked with both unawareness of situation and the intensity of visual stimulation.

The first group is characterised with significant gaps (p<0.05) between ‘Alarm’ and the other two scenarios, but not between ‘Smoke’ and ‘Flame’. A greater rise in the self-evaluated score of surprise is seen with unawareness of situation, while a greater drop in dominance is observed. A clearer situation brings a smaller amplitude of pupil dilation and spatially less random allocation of gaze, whilst an unclear situation induces larger pupil dilation, but does not escalate the spatial randomness of gaze.

The second group features significant gaps between ‘Smoke’ and the other two scenarios. The ‘Smoke’–‘Alarm’ difference has a connection with unawareness of situation, while the ‘Smoke’–‘Flame’ difference is related to the intensity of visual stimulation. The ‘Alarm’–‘Flame’ difference in this case, however, is not necessarily significant. The self-evaluated scores of fear and arousal rise with both factors. The time ratio of saccade holds a positive relation with unawareness of situation, and a negative relation with the intensity of visual stimulation, while an opposite pattern is found with the time ratio of fixation. Both factors are positively related to the brain activation coefficients ($\hat{\beta }$) of all 8 channels that were measured at.

The findings of this study indicate that the research on panic during fire evacuation can be broken down into multiple sub-elements that may have various impacts on the psycho- and physiological changes of evacuees. These sub-elements include at least the unawareness of situation and the intensity of visual stimulation. By taking into account the sub-elements of panic, it is possible to extend evacuation–panic-related researches to a more refined level that potentially leads to more accurate and interpretable discoveries. This possibility applies to both experiments and model simulations in a sense that future researches could base the experiment design on the panic mechanism that is unveiled, and further reasonably parametrise panic in pedestrian models. It is also suggested in a practical sense that, faced with a real-world fire, apart from containing the disaster itself, it may be helpful to inform the evacuees with adequate information. An efficient evacuation reduces the exposure to the hazard source, and therefore reduces the overall risk. Additionally, it is reasonable to believe that exercises and drills contribute to increased preparedness of the general public, which may escalate their awareness when faced with emergency situations, and eventually reduce the risk.

## Figures and Tables

**Table 1:** Mean values of basic emotion shifts.

Scenario	Sadness	Happiness	Fear	Anger	Surprise	Disgust
Alarm	1.47	−1.47	3.67	1.07	2.24	1.78
Smoke	1.02	−1.20	2.14	0.67	1.18	1.25
Flame	1.24	−1.36	2.49	0.76	1.29	1.40

**Table 2:** Standard deviations of basic emotion shifts.

Scenario	Sadness	Happiness	Fear	Anger	Surprise	Disgust
Alarm	1.68	1.73	1.73	1.56	2.28	2.02
Smoke	1.27	1.64	1.65	1.29	1.93	1.69
Flame	1.56	1.59	1.86	1.37	2.06	1.95

**Table 3:** *p*-Values of basic emotion shifts.

Contrast	Sadness	Happiness	Fear	Anger	Surprise	Disgust
Alarm-Smoke	**0.002**	**0.010**	**0.000**	**0.000**	**0.000**	**0.001**
Alarm-Flame	0.129	0.293	**0.000**	**0.010**	**0.000**	**0.018**
Smoke-Flame	**0.044**	**0.011**	**0.031**	0.229	0.444	0.132

**Table 4:** Mean values of SAM shifts.

Scenario	Valence	Arousal	Dominance
Alarm	−2.05	3.73	−1.38
Smoke	−1.79	2.35	−0.80
Flame	−1.91	2.64	−0.86

**Table 5:** Standard deviations of SAM shifts.

Scenario	Valence	Arousal	Dominance
Alarm	1.34	2.00	1.78
Smoke	1.20	1.79	1.77
Flame	1.38	1.96	1.69

**Table 6:** *p*-values of SAM shifts.

Contrast	Valence	Arousal	Dominance
Alarm-Smoke	**0.019**	**0.000**	**0.001**
Alarm-Flame	0.316	**0.000**	**0.002**
Smoke-Flame	0.118	**0.034**	0.614

**Table 7:** Mean values and standard deviations of pupil dilation.

Scenario	μ	σ
Alarm	1.46	0.24
Smoke	1.31	0.14
Flame	1.32	0.18

**Table 8:** *p*-values of pupil dilation.

Contrast	Alarm-Smoke	Alarm-Flame	Smoke-Flame
*p*-value	**0.000**	**0.000**	0.543

**Table 9:** Mean values of $r_{\mathrm{fixation}}$ and $r_{\mathrm{saccade}}$.

Scenario	$r_{\mathrm{fixation}}$	$r_{\mathrm{saccade}}$
Roaming	0.863	0.052
Alarm	0.835	0.063
Smoke	0.887	0.040
Flame	0.903	0.027

**Table 10:** Standard deviations of $r_{\mathrm{fixation}}$ and $r_{\mathrm{saccade}}$.

Scenario	$r_{\mathrm{fixation}}$	$r_{\mathrm{saccade}}$
Roaming	0.087	0.017
Alarm	0.101	0.022
Smoke	0.099	0.024
Flame	0.085	0.023

**Table 11:** *p*-Values of $r_{\mathrm{fixation}}$ and $r_{\mathrm{saccade}}$.

Contrast	$r_{\mathrm{fixation}}$	$r_{\mathrm{saccade}}$
Roaming-Alarm	**0.018**	**0.002**
Roaming-Smoke	**0.031**	**0.003**
Roaming-Flame	**0.000**	**0.000**
Alarm-Smoke	**0.000**	**0.000**
Alarm-Flame	**0.000**	**0.000**
Smoke-Flame	0.056	**0.007**

**Table 12:** Mean values and standard deviations of binned entropy of gaze.

Scenario	μ	σ
Roaming	4.34	0.33
Alarm	4.25	0.47
Smoke	3.57	0.64
Flame	3.66	0.67

**Table 13:** *p*-values of binned entropy of gaze.

Contrast	*p*-Value
Roaming-Alarm	0.169
Roaming-Smoke	**0.000**
Roaming-Flame	**0.000**
Alarm-Smoke	**0.000**
Alarm-Flame	**0.000**
Smoke-Flame	0.299

**Table 14:** Mean values of activation coefficient.

Scenario	Ch-1	Ch-2	Ch-3	Ch-4	Ch-5	Ch-6	Ch-7	Ch-8
Alarm	5.71	5.32	5.01	4.46	5.18	5.40	5.52	5.04
Smoke	3.13	3.14	2.88	2.91	3.20	2.36	2.79	2.09
Flame	4.85	4.14	4.32	3.77	4.44	4.31	3.68	3.42

**Table 15:** Standard deviations of activation coefficient.

Scenario	Ch-1	Ch-2	Ch-3	Ch-4	Ch-5	Ch-6	Ch-7	Ch-8
Alarm	2.69	2.56	1.55	1.68	2.92	2.03	1.94	1.91
Smoke	1.89	2.10	1.87	2.01	1.71	1.78	1.59	1.37
Flame	2.51	1.83	2.74	1.74	2.19	1.81	2.00	1.00

**Table 16:** *p*-values of activation coefficient.

Contrast	Ch-1	Ch-2	Ch-3	Ch-4	Ch-5	Ch-6	Ch-7	Ch-8
Alarm-Smoke	**0.000**	**0.000**	**0.000**	**0.000**	**0.001**	**0.000**	**0.000**	**0.000**
Alarm-Flame	0.133	**0.000**	0.164	**0.009**	0.196	**0.001**	**0.000**	**0.000**
Smoke-Flame	**0.000**	**0.001**	**0.002**	**0.003**	**0.001**	**0.000**	**0.011**	**0.000**

## Data Availability

The data presented in this study are available on request from the corresponding author. The data are not publicly available due to privacy concerns of subjects.
